# Colo-cutaneous fistula to the thigh secondary to acute sigmoid diverticulitis: a case report

**DOI:** 10.1093/jscr/rjad267

**Published:** 2023-05-18

**Authors:** Eduardo Serpa, Felipe Pacheco, Sundarachalam Pindicura, Omar Marar

**Affiliations:** Department of Surgery, Central Michigan University College of Medicine, Saginaw MI 48601, United States; Department of Surgery, Central Michigan University College of Medicine, Saginaw MI 48601, United States; Department of Surgery, Central Michigan University College of Medicine, Saginaw MI 48601, United States; Department of Surgery, Central Michigan University College of Medicine, Saginaw MI 48601, United States

## Abstract

Acute diverticulitis is one of the most common gastrointestinal illnesses that requires hospital admission. It has a broad range of presentations from uncomplicated disease to perforation and peritonitis that require emergent surgical exploration. Abscesses are one of the most common complications. We present a case of retroperitoneal abscess with extension to the antero-lateral upper thigh that was successfully treated with open Hartman’s procedure with drainage of psoas abscess and open drainage of thigh abscess.

## INTRODUCTION

In the United States, acute diverticulitis represents the third most common gastrointestinal illness that require hospitalization [1]. Computed tomography (CT) scan is the modality of choice to assess for complications and rule out other possible diagnosis. Acute complicated diverticulitis requires management of both colonic inflammation, and the specific complications. Depending on the size and location, diverticular abscesses are usually amenable for drainage [2]. We present a case of complicated diverticulitis with psoas abscess that failed drainage with subsequent fistulization to the left thigh.

## CASE REPORT

**Image 1:**
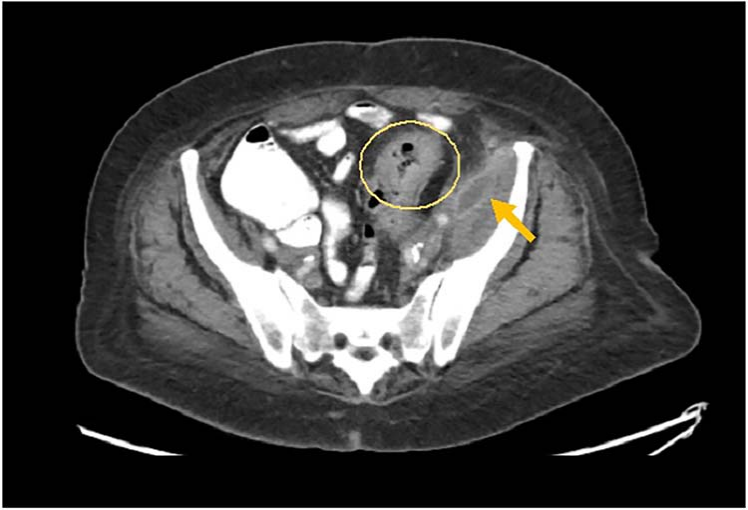
Arrow: psoas abscess. Circle: inflamed colon.

**Image 2:**
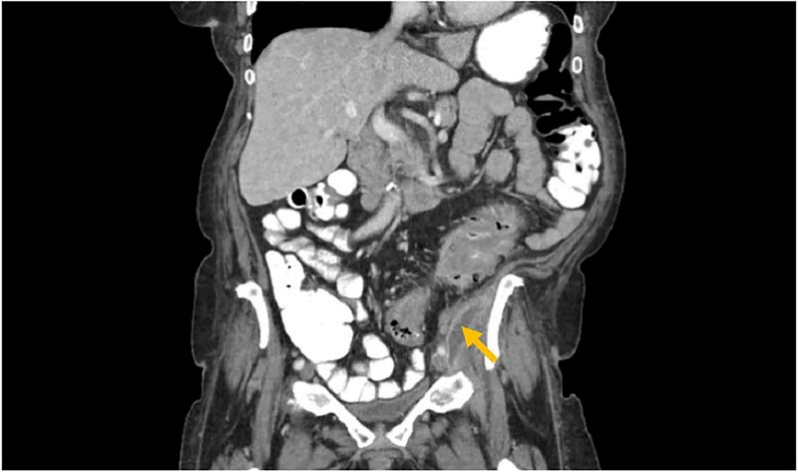
Arrow: psoas abscess.

A 73-year-old female with past medical history of hypertension, diabetes mellitus, hyperlipidemia, recent history of complicated sigmoid diverticulitis with psoas abscess measuring 1.4 cm × 6.5 cm (Images 1 and 2). Patient underwent interventional radiology drain placement in the abscess cavity and antibiotic treatment with adequate response and was discharged home with oral antibiotics. Her last colonoscopy was 1 year prior, with no evidence of polyps. She followed up after 10 days with our colorectal surgeon. She denied any pain, her drain output was minimal. She was offered surgical intervention, however she refused. Drain was removed. She presented to the hospital again after 3 weeks with lower abdominal pain with radiation to left back and with anterior left upper thigh pain. She felt a sensation of fullness in that area. She was tolerating diet and having normal bowel movements. She denied any fevers, chills, urinary complaints. On the physical exam, her abdomen was soft, non-distended, there was tenderness to palpation in her left lower quadrant, no rebound, no rigidity. There was also severe tenderness to palpation in the left antero-lateral thigh. There were no skin changes, crepitus to palpation or drainage from this area.

**Image 3:**
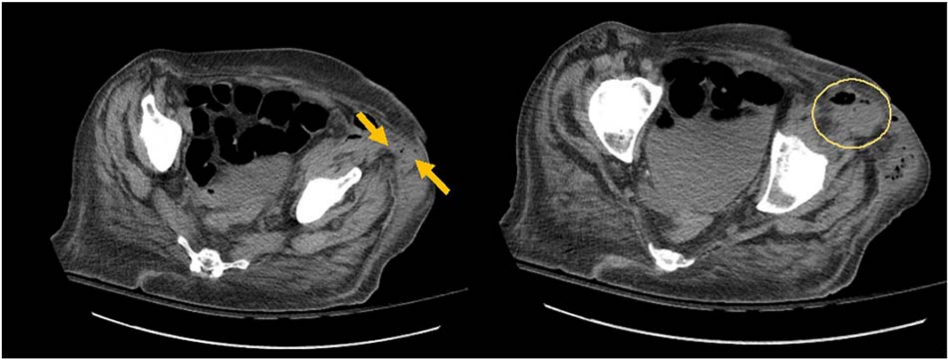
Arrows shows extension of psoas abscess through abdominal wall in the surrounding tissues around the hip. There is not Hip bone involvement. Circle shows abscess next to the hip.

The patient had leukocytosis of 18.23 (Normal: 3.4–11 k/cmm) and CT scan of the abdomen pelvis without Intravenous contrast showed sigmoid diverticulitis with partial resolution of psoas abscess. However, it revealed a tract from the abscess to the hip (Image 3). After a prolonged discussion with the patient, she agreed to proceed with surgical intervention. She underwent exploratory laparotomy. There was a firm, inflamed sigmoid colon densely adherent to the psoas muscle. She also had two loops of small bowel that were densely adhered to the colon. Those loops were removed in block and two small bowel anastomoses were created. After mobilization of the sigmoid colon, there was a defect with fistulization to the psoas muscle. There was a significant amount of purulent fluid coming out of the psoas muscle, which was copiously suctioned and irrigated. Then, an end colostomy was created. A 3 cm elliptical incision was made in the left thigh and the skin was removed. The tissue was probed digitally, and we were able to identify the tract with a significant amount of stool and purulent fluid emanating from the left thigh. Once the cavity was entered, pressure was applied on the thigh, the psoas abscess was draining as well, indicating communication. We drained as much as we possibly could through both the psoas and the left thigh abscess and irrigated them copiously and packed. The patient was hemodynamically stable during the procedure and was extubated and transferred to the floor. The nasogastric tube was removed on post operative day 2, and she started on a diet. Her left hip dressing was changed daily with no signs of further infection. She continued on antibiotics during all hospital stay. She was discharged to Nursing home for physical therapy and adequate wound care. Specimen pathology was negative for malignancy. Patient was successfully reversed 6 months later.

## DISCUSSION

Diverticulitis is classified as complicated and uncomplicated according to findings in CT scan. Recently, for selected patients with noncomplicated diverticulitis, antibiotics have no proven benefit. On the other hand, in complicated diverticulitis, antibiotics are indicated. The presence of abscess in CT scan indicates complicated disease. Depending on the size and location of the abscess, drainage is indicated. If this fails, surgical intervention is required [3]. Psoas abscess can arise via contiguous spread from adjacent structures such as diverticulitis, or by hematogenous route from a distant site. This abscess can extend distally and present as a painful or painless mass below the inguinal ligament. The treatment of choice for psoas abscess is percutaneous drainage, which is successful in 90% of the cases. The drain is usually removed after drainage has ceased. If percutaneous drainage fails, surgical drainage is indicated, either laparoscopic or open has been shown to be successful [4]. There are some reports of acute complicated diverticulitis that presented as groin abscess, which were addressed with Hartman’s procedure and drainage of the abscess cavity [5]. Our patient failed conservative management with percutaneous drainage of acute diverticulitis, most likely due to persistent inflammation and presence of a large fistula. She presented with psoas abscess tracking down to the thigh. We performed open Hartman’s procedure with drainage of psoas abscess and external drainage of the abscess. Post-operatively our patient did not develop wound complications. She was successfully reversed 6 months later. We conclude that this combined approach is important in the treatment of these abscesses to prevent recurrence.

## References

[1] Almario CV , Ballal ML, Chey WD, Nordstrom C, Khanna D, Spiegel B. Burden of gastrointestinal symptoms in the United States: results of a nationally representative survey of over 71,000 Americans. Am J Gastroenterol 2018;113:1701–10. 30323268 10.1038/s41395-018-0256-8 PMC6453579

[2] Sugi MD , Sun DC, Menias CO, Prabhu V, Choi HH. Acute diverticulitis: key features for guiding clinical management. Eur J Radiol 2020;128:109026. Epub 2020 Apr 30PMID: 32422553. 32422553 10.1016/j.ejrad.2020.109026

[3] You H , Sweeny A, Cooper ML, Von Papen M, Innes J. The management of diverticulitis: a review of the guidelines. Med J Aust 2019;211:421–7 Epub 2019 Jul 28PMID: 31352692. 31352692 10.5694/mja2.50276

[4] Spelman D . Psoas abscess. UpToDate, June 2022. https://www.uptodate.com/contents/psoas-abscess?search=psoas+abscess&source=search_result&selectedTitle=1∼31&usage_type=default&display_rank=1.

[5] Ruscelli P , Renzi C, Polistena A, Sanguinetti A, Avenia N, Popivanov G, et al. Clinical signs of retroperitoneal abscess from colonic perforation: two case reports and literature review. Medicine (Baltimore) 2018;97:e13176. PMID: 30407351PMCID: PMC6250550. 30407351 10.1097/MD.0000000000013176 PMC6250550

